# Publisher Correction: Rotating robots move collectively and self-organize

**DOI:** 10.1038/s41467-018-03873-x

**Published:** 2018-04-11

**Authors:** Christian Scholz, Michael Engel, Thorsten Pöschel

**Affiliations:** 1 Institut für Multiscale Simulation, Friedrich-Alexander-Universität Erlangen-Nürnberg, 91052 Erlangen, Germany; 2 Institut für Theoretische Physik II: Weiche Materie, Heinrich-Heine-Universität Düsseldorf, 40225 Düsseldorf, Germany

Correction to: *Nature Communications* 10.1038/s41467-018-03154-7, published online: 02 Mar 2018

The original version of this Article contained an error in Fig. 5. In Fig. 5e, the scale on the *y*-axis originally incorrectly went form ‘0’ to ‘1.10^−4^’. The correct scale goes from ‘0’ to ‘0.4’. In Fig. 5f, the *x*-axis labels were incorrectly shifted to the right. The correct version of Fig. 5 is:

which replaces the previous:

This has been corrected in both the PDF and HTML versions of the Article.

