# Supplementary material for: Rotating robots move collectively and self-organize
Source: Nat Commun. 2018 Mar 2;9:931. doi: 10.1038/s41467-018-03154-7 (PMC5834624; doi:10.1038/s41467-018-03154-7)
Supplement: Supplementary file 1 — Supplementary Information [file 41467_2018_3154_MOESM1_ESM.pdf]

—Supplementary Information— Rotating Robots Move Collectively and Self-Organize

Christian Scholz,[1,2,*] Michael Engel,[1,†] and Thorsten Pöschel[1,‡]

[1]*Institute for Multiscale Simulation, Friedrich-Alexander-University Erlangen-Nürnberg, 91052 Erlangen, Germany*
[2]*Institut für Theoretische Physik II: Weiche Materie, Heinrich-Heine-Universität Düsseldorf, D-40225 Düsseldorf, Germany*

(Dated: January 20, 2018)

* christian.scholz@hhu.de
† michael.engel@fau.de
‡ thorsten.poeschel@fau.de

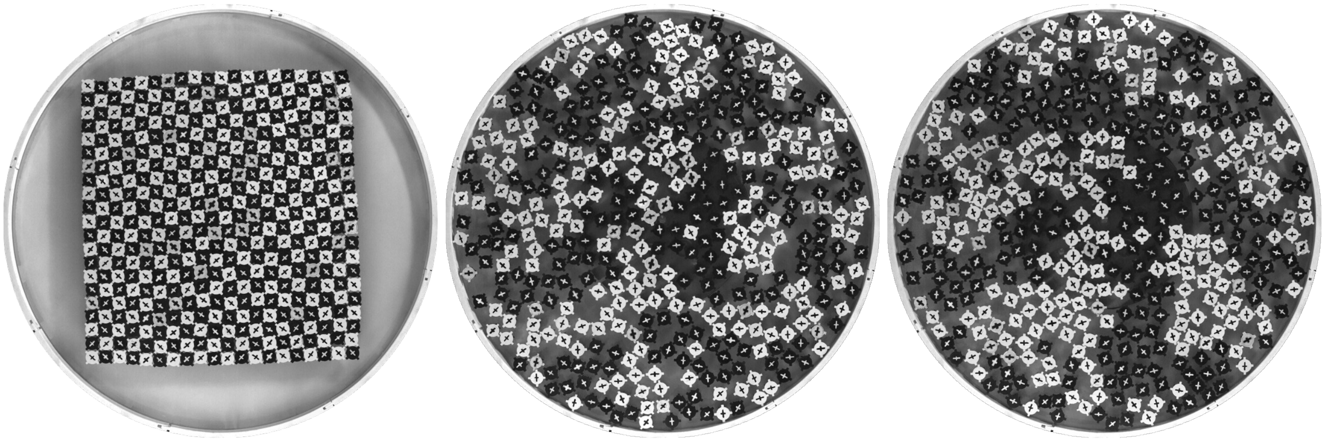

Supplementary Figure 1. Snapshots from Supplementary Movie 1 at times 0 s, 30 s and 60 s.

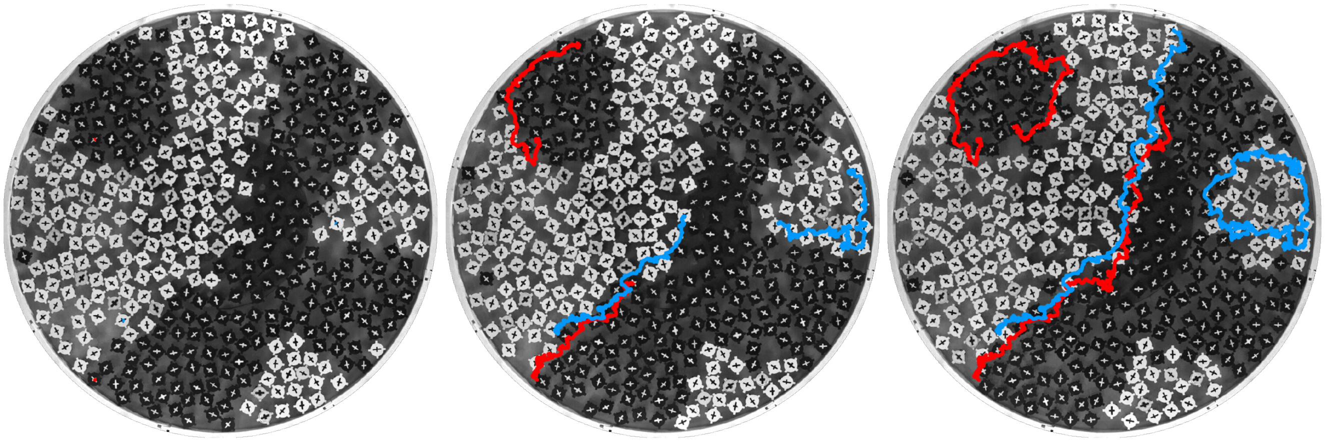

Supplementary Figure 2. Snapshots from Supplementary Movie 2 at times 0 s, 26 s and 52 s.

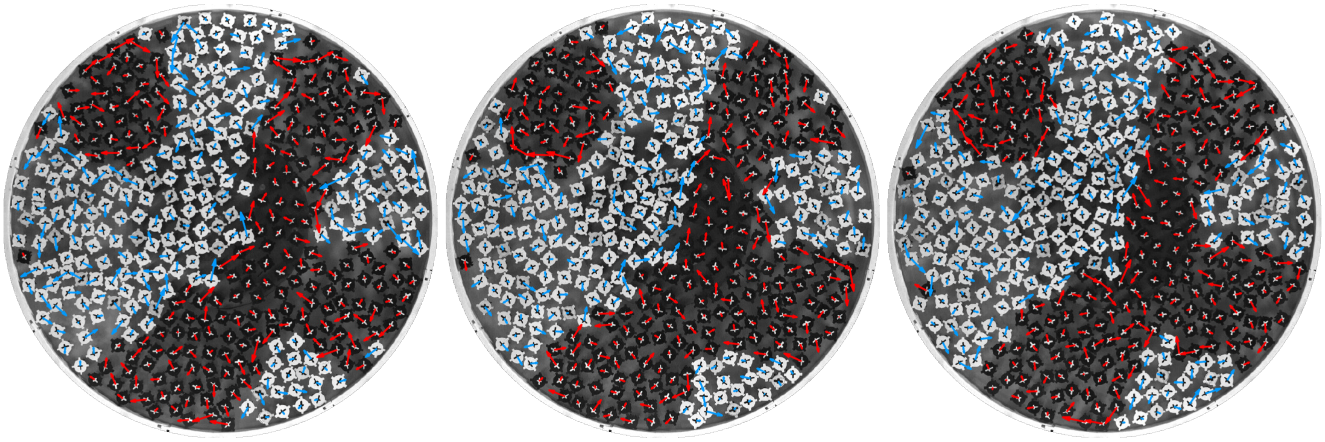

Supplementary Figure 3. Snapshots from Supplementary Movie 3 at times 0 s, 13 s and 26 s.

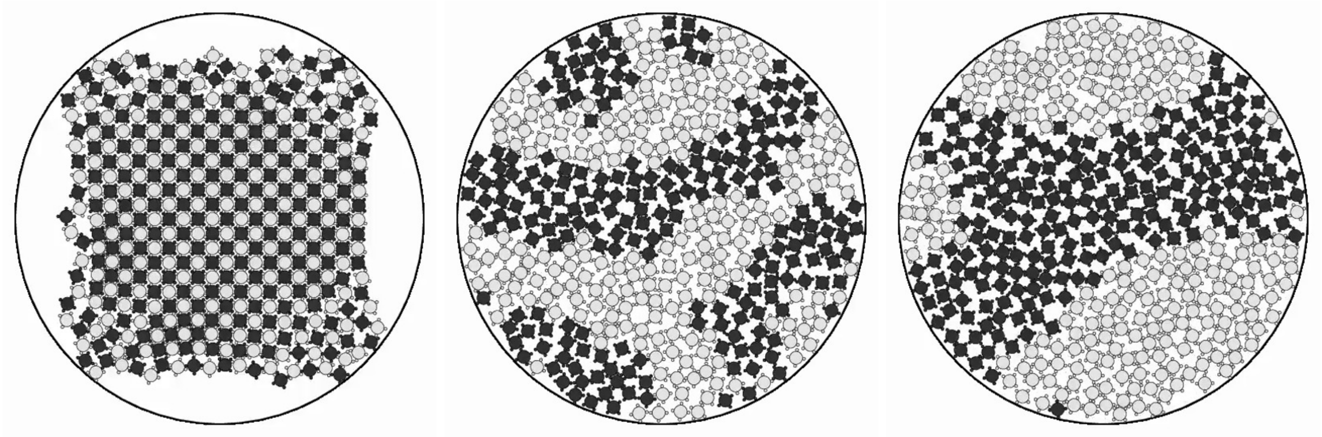

Supplementary Figure 4. Snapshots from Supplementary Movie 4 (after 0 s, 100 s, 1000 s real time).

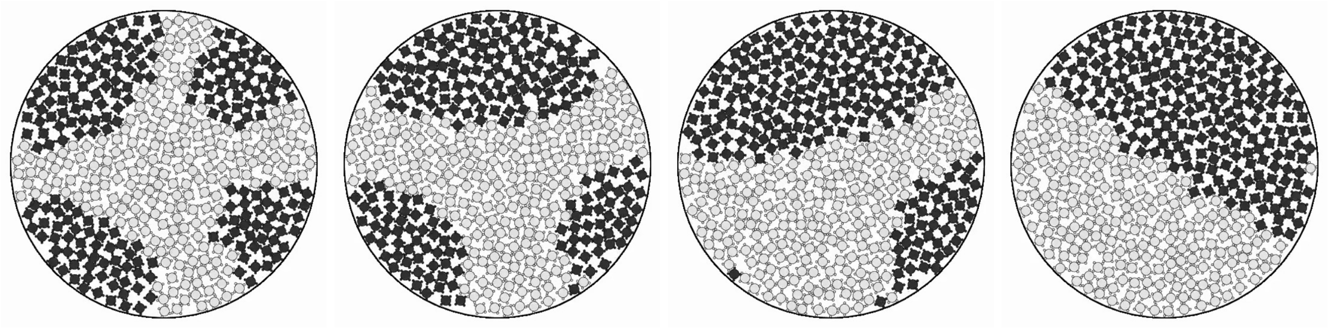

Supplementary Figure 5. Snapshots from Supplementary Movie 5 (after 500 s, 1000 s, 2000 s, 4000 s real time).

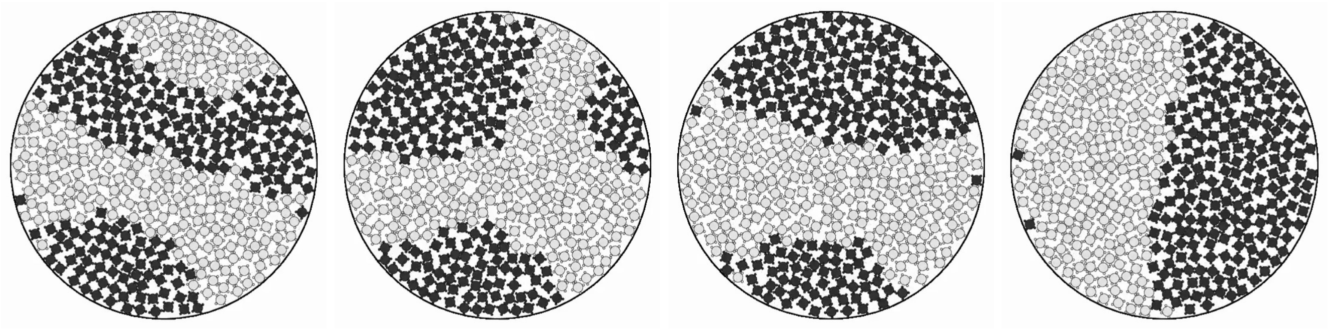

Supplementary Figure 6. Snapshots from Supplementary Movie 6 (after 500 s, 2000 s, 3500 s, 7000 s real time).
