# Supplementary material for: Rotating robots move collectively and self-organize
Source: Nat Commun. 2018 Mar 2;9:931. doi: 10.1038/s41467-018-03154-7 (PMC5834624; doi:10.1038/s41467-018-03154-7)
Supplement: Supplementary file 3 — Description of Additional Supplementary Files [file 41467_2018_3154_MOESM3_ESM.pdf]

Description of Additional Supplementary Files

File Name: Supplementary Movie 1
Description: Initial Segregation
Experimental demonstration of demixing of a binary mixture of rotors from the initial state in real time.

File Name: Supplementary Movie 2
Description: Interface Motion
Trajectories of two clockwise and two anti-clockwise spinning rotors at interfaces in real time.

File Name: Supplementary Movie 3
Description: Particle Velocities
Velocities of particles in the demixed state in real time.

File Name: Supplementary Movie 4
Description: Demixing in simulations
Langevin dynamics simulation of the demixing process. Video is time lapsed by a factor of 15.

File Name: Supplementary Movie 5
Description: Metastable states (four- and three-fold)
Langevin dynamics simulation showing four- and three-fold symmetric metatable states. Video is time lapsed by a factor of 150.

File Name: Supplementary Movie 6
Description: Metastable states (two- and three-fold)
Langevin dynamics simulation showing two- and three-fold symmetric metatable states. Video is time lapsed by a factor of 150.

File Name: Supplementary Data 1
Description: Langevin dynamics simulation code
C++ code of the Langevin dynamics simulations.

File Name: Supplementary Data 2
Description: Rotor CAD file
OpenSCAD file describing the geometry of the rotor.

File Name: Supplementary Data 3
Description: Rotor STL file
STL geometry file of the rotor.
